# Knowledge and associated factors of lactational amenorrhea as a contraception method among postpartum women in Aksum town, Tigray Region, Ethiopia

**DOI:** 10.1186/s13104-018-3754-2

**Published:** 2018-09-03

**Authors:** Teklehaymanot Huluf Abraha, Alemayehu Shimeka Teferra, Abebaw Addis Gelagay, Tsehaynesh Gidey Welesamuel, Gezienesh Kahsay Fisseha, Berihu Gidey Aregawi, Desta Siyoum Belay

**Affiliations:** 1 School of Public Health, College of Health Sciences, Aksum University, P.O.Box: 1010, Aksum, Tigray Ethiopia; 2 Institute of Public, College of Medicine and Health Sciences, University of Gondar, Gondar, Ethiopia; 3 School of Nursing, College of Health Sciences, Mekelle University, Mekelle, Tigray Ethiopia

**Keywords:** Lactational amenorrhea method, Knowledge, Contraception, Aksum, Tigray, Ethiopia

## Abstract

**Objectives:**

The objective of the study was to assess the prevalence of knowledge level and predictors of lactational amenorrhea method (LAM) as method of contraception among women who gave birth a year prior to the study period in the Aksum town, Tigray Region. The study was cross sectional in design conducted from March 25 to April 24, 2015. Results of the study could help the design of family planning strategies.

**Results:**

The knowledge status of LAM as a contraceptive method was 8.8% [95% CI 6.4–11%)]. Women who delivered at health institution (AOR = 1.4, 95% CI 1.2–4.3), attended postnatal care (AOR = 1.3, 95% CI 1.2–3.0) and visited home and counseled about family planning by health extension in the last 12 months, (AOR = 1.5, 95% CI 1.3–4.0) were more likely found knowledgeable towards LAM. Secondary and above level of the maternal education was also found a significant predictor variable with LAM as a contraceptive method (AOR = 1.2 95% CI 1.1–4.0). Our findings recommend that to address the knowledge gap of mothers; improving the uptake of maternal health services and strengthening family planning counseling at home are a key area for improving the knowledge level of LAM.

**Electronic supplementary material:**

The online version of this article (10.1186/s13104-018-3754-2) contains supplementary material, which is available to authorized users.

## Introduction

The lactational amenorrhea method (LAM) is a highly effective for preventing subsequent pregnancy (98%) during the first 6 months postpartum method of contraception for postpartum women [1–5]. The development of LAM began in 1988 when a group of experts from around the world met in Bellagio, Italy to define a set of guidelines that a woman could use to predict her return to fertility during breast feeding [6]. LAM should have three criteria to be used; exclusively breast feeding a child who is less than 6 month old and mother should be amenorrhea [2, 3].

Worldwide, more than 90 percent of women during the post partum period desire either to delay or limit their next pregnancies [7]. Most of postpartum women want to prevent pregnancy during first 2 years after delivery, but had not yet received any contraceptive method [8].

In Ethiopia, maternal health problems remain a major public health problem since pregnancy and child birth are the leading cause of mortality and morbidity among the sexually active women [9, 10]. Half of all non first pregnancies occur less than 1 year following preceding birth [11]. At national level, there is high level of unmet need for family planning in the first year following delivery (i.e. 80%) [12]. LAM is a natural defense against pregnancy, in expensive contraceptive, safe for mothers [2], and provide ideal nutrition and defense against infection and disease for infants [5]. It increases a subsequent adoption of other modern contraceptive methods. Since counseling on LAM includes information on transition to other modern contraceptive methods once LAM criteria are no longer met, LAM has the potential to increase uptake of contraception after is no longer effective [5, 6, 13, 14].

Despite the benefit provided by exclusive breast feeding to women as an important condition to LAM and for the survival of infants, and increasing knowledge regarding LAM as a method of contraception and we can simply avert unwanted pregnancy by adopting LAM [5, 6]. However; LAM in Ethiopian Health Sector Transformation Plan (EHSTP) agenda has less emphasized [10]. To our knowledge; the LAM knowledge status in Ethiopia particularly in urban community has not been studied, where fertility rate is generally high [9] and health resources extremely limited [10]. We therefore; sought to assess the knowledge level and predictors of LAM as method of a contraception among women gave birth in the past 1 year prior to the study period. This could help family planning planners to develop strategies for the prevention of closely spaced, unintended pregnancies and maximizing use of LAM as a method of contraceptive method in the first 6 months.

## Main text

### Methods

#### Study design and period

This was a community based cross sectional study survey done in the Aksum town, Tigray Region, northern Ethiopia, from March 25 to April 24, 2015.

#### Study setting

Aksum town is the study area. It is located 1067 km to the north of Addis Ababa, the capital city of Federal Democratic Republic of Ethiopia, and 248 km from Mekelle city of Tigary Regional State. According the town administrative health office the estimated total population 60,706, of whom 30,960 are women living in five *Kebeles.* Majority of the population depends on none agriculture production. The town has achieved universal health coverage. There are two public health centers, one referral hospital, one zonal hospital, one Family Guidance Association of Ethiopia clinic, five private clinics and ten drug shops providing maternal, child and other health services to the population [15].

#### Sources population

All reproductive age women who gave birth in the last 12 months prior to the study period (March 25, 2014–March 25, 2015) who living in the town of Aksum were taken as sources population.

#### Study population

All reproductive age women who gave birth in the last 12 months who living in the town of Aksum during the data collection period.

#### Sample size determination and procedures

A single sample proportion formula was used to determine the sample size considering the following assumptions: Since there is no a study done in the study area or Ethiopia, the proportion women who have a knowledge on LAM in extended postpartum period was assumed to be 50%, 95% confidence intervals, 5% absolute level of precision [16], and 1.5% design effect considered. In addition a none response rate 5% was utilized and finally a sample size of 604 was determined.

#### Sampling technique

The study population was selected using multistage sampling technique. Two stage sampling was used to select the study participants. At the first stage by using simple random sampling two *Kebeles* was selected from four total *Kebeles.* At the second stage study participants was selected systematically after allocating the total number of postpartum women (sample) to each of the selected *Kebeles* proportionally. The study participants were selected by systematic random sampling techniques before interview. Before the actual data collection; community-based survey was done for 7 day to trace the postpartum women (Additional file 1: Figure S1).

#### Operational definitions

*Knowledge of LAM as a contraception method* refers to the study participants’ spontaneously mentioned the following three criteria: (a) feeding exclusively with mother’s milk; (b) postpartum amenorrhea; (c) infant younger than six months [3, 5, 17]. A binary dependent variable indicating if the study participants mentioned all the above three criteria, it categorized as knowledgeable (1), if not (0).

*Antenatal care services use* proportion of women who have received antenatal care at least one and above visit either at health center or hospital.

*Fertility desire* This was defined as the need to have another child in the future as expressed at the time of data collection [18]. The other detail measurements variables are indicated in Additional file 2: Table S1.

#### Data collection and quality control

Data was collected via face to face interview at the study participant’s home using a structured and pre-tested questionnaire. The tool was prepared originally in English and translated to ‘’*Tigrigna’’* and translated back to English in order to maintain internal consistency. Four female diploma midwife holders and a Bachelor of Science in nurse resident supervisor were involved during the data collection period.

#### Data processing and analysis

Data were entered using EPI INFO version 7 and exported to STATA version 12 for analysis. Descriptive statistics were presented in the form of tables and texts. To identify factors associated with knowledge of LAM as a method contraceptive, binary logistic regression analysis was conducted. P-value < 0.05 and 95% CI for adjusted odds ratios were used to confirm the statistical significance of the associations [16].

### Results

#### Socio-demographic characteristics

In this study, 604 postpartum women were interviewed. From these, 590 (97.7%) women completely responded to the questioner. The mean age of the study samples was 27.4 ± 5.0 years. Two hundred thirty-one (39.2%) were aged between 25 and 29 years; 3.2% were teenagers (< 20 years). Three hundred eighty-three (64.9%) were housewives. Majority (52.2%) of the study participants had attended secondary and above educational level and 38.5% of their partner attended primary educational school (Table 1).

**Table 1:** Socio-demographic variables in Aksum town, Tigray Region, northern Ethiopia, June 2015

Variables	Frequency (n)	Percentage (%)
Age		
16–19	19	3.2
20–24	149	25.2
25–29	231	39.2
30–34	126	21.4
≥ 35	65	11.0
Marital status		
Married	543	92.0
Others^a^	47	8.0
Educational level		
No formal education	80	13.6
Primary education	202	34.2
Secondary education and above	308	52.2
Partner’s education (n = 545)		
No formal education	24	4.4
Primary school	210	38.5
Secondary school	185	34.0
Tertiary school	126	23.1
Occupation		
House wife	383	64.9
Government employee	46	7.8
Private employee	103	17.5
Daily labourer	43	7.3
Others^b^	15	2.5
Partner occupation (n = 545)	125	22.9
Government employee		
Private employee	257	47.2
Daily labourer	131	24.0
Others^c^	32	5.9

^a^ Single, separated, divorced, or widowed, ^b^ Alcohol/Swa seller, farmer, or student, ^c^ Farmer, pension, or guard

#### Maternal health services use related characteristics

In this study a total 579 or 98.1% of the study participants attended at least one antenatal care visits. Five hundred twenty-four of the women had received four or more ANC visits. Five hundred seventy-seven delivered by skill attendants at health institution. Two hundred fifty eight (43.7%) had received postnatal care follow up either at health center or hospital (Additional file 3: Table S2).

#### Knowledge of lactational amenorrhea as a contraceptive method during postpartum period

In this study, the knowledge of lactation amenorrhea as a contraceptive method was found to be fifty-two (8.8%) [95% CI (6.4–11%)].

#### Knowledge sources on lactational amenorrhea as a contraception method

**Fig. 1:**
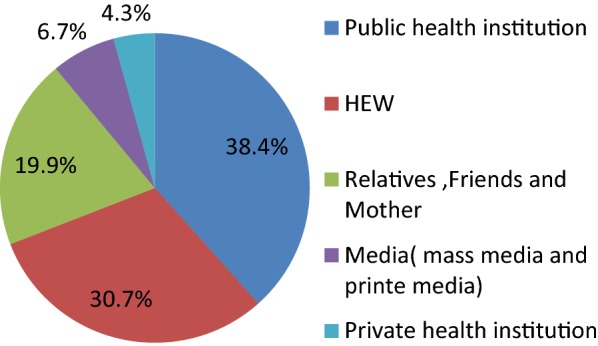
Knowledge sources on lactational amenorrhea as a contraception method in Aksum town, Tigray Region, northern Ethiopia, June 2015

Public health institution (38.4%), health extension worker (30.7%), relatives, friends and mothers (19.9%), media (mass media, and print media) (6.7%) and private health institution (4.3%) (Fig. 1).

#### Factors associated with knowledge of lactational amenorrhea as a contraceptive method

During the multivariable logistic regression model, maternal educational level, place of birth, postnatal care, visited and counseled for family planning by health extension workers in the last 12 months were found significantly associated with the outcome variable when we adjusted for variables P-value less than 0.2.

Participates who gave birth at health institution were 1.4 times (AOR = 1.4, 95% CI 1.3–4.3) more likely have found a sufficient knowledge on lactational amenorrhea method as a method contraceptive than as compare to those who gave birth at home. Women who had postnatal care visit were 1.3 times (AOR = 1.3 95% CI 1.2–3.0) more likely knowledgeable about lactational amenorrhea method as a method of contraception in the extended postpartum period than as compare to those who have no follow up services. Those women who had visited home and counseled for family planning by health extension worker in the last 12 months were 1.5 times (AOR = 1.5, 95% CI 1.3–4.0) more likely to increase knowledge toward lactational amenorrhea method as compare to women who did not visited and counseled about family planning at their home by health extension worker. In addition, study participants with secondary and above maternal education were more likely to be knowledgeable than women with primary and no formal education (AOR = 1.2, 95% CI 1.1–4.0) (Table 2).

**Table 2:** Predictors of lactational amenorrhea method knowledge of postpartum women in Aksum town, Tigray Region, Ethiopia, June 2015

Variable	Knowledge of LAM				
	Yes	No	COR (95% CI)	AOR (95% CI)	P-value
Maternal educational attainment					
No formal education	5	75	Ref	Ref	
Primary educational	24	178	0.82 (0.3–2.24)	0.6 (0.1–3.0)	
Secondary education and above	23	285	1.67 (1.2–3.0)^a^	1.2 (1.1–4.0)^a^	0.00
Place of birth					
Home	1	12	Ref	Ref	
Health institution	51	526	1.16 (1.11–9.13)^a^	1.4 (1.2–4.3)^a^	0.04
PNC					
Yes	21	237	1.16 (1.13–2.0)^a^	1.3 (1.2–3.0)^a^	0.02
No	31	301	Ref	Ref	
Visited and counseled FP by HEW in the last 12 months					
Yes	42	415	1.14 (1.1–2.5)^a^	1.5 (1.3–4.0)^a^	0.001
No	10	123	Ref	Ref	
Fertility desire					
Yes	42	444	Ref	Ref	
No	10	94	1.12 (1.1–2.3)^a^	1.02 (0.30–2.81)	0.13
Number of live children					
1	10	172	Ref	Ref	
2–3	30	251	0.55 (0.23–1.3)	0.4 (0.3–1.7)	
≥ 4	12	115	1.14 (1.1–2.3)^a^	1.7 (1.2–7.0)	0.82

*FP* family planning, *HEW* health extension worker, *PNC* postnatal care, *Ref* reference category

^a^Statistically associated

### Discussion

There are a clear evidence show that; lactational amenorrhea method is highly effective contraceptive method for fertility control when the mother is well informed and supported how to use as method of contraception [19]. LAM for postpartum mothers can be used effectively and reliably as a method of contraception [3]. This study is the first to assess the level of knowledge LAM as a method of contraceptive and its determinants in Ethiopia. Therefore, identifying the knowledge gap and potential factors associated with it; is an important step to improve the adoption rate of lactational amenorrhea method as a contraception option and it can help to improve exclusive breast feeding practice [20], in the region particularly in Ethiopia.

This finding show that the knowledge level on lactational amenorrhea method was found 8.8% (95% CI 6.4–11%); this finding higher than a study done in Cairo (1%) [21]. This might be attributing to the coordinated efforts done by health workers and health extension workers of house to house based health education on maternal health servicers’ use.

In this study, study participants with secondary and above educational level were 1.2 times more likely to have knowledge than study participants with no formal and primary education. This statistically significance and positive relationship could be explained by the fact that educated mothers are more knowledgeable on the importance LAM; and also educated mothers are more likely to visit health institution and they may have access to media and written information on lactational amenorrhea method. This association is line with finding reported a study done in eastern Turkey [22].

Place of birth (health facility versus home) and postnatal care services use was found important predictor associated with knowledge of LAM. The possible explanation is study participants who delivered at health facility and attended postnatal care might get an opportunity comprehensive counseling about the modern and traditional contraceptive during their maternal health services utilization. This is line with a study conducted in Niger [23].

In line with previous study done in Niger [23], mothers visited and counseled for family planning by town HEW in the last 12 months prior the study at their home showed an independent associated with the lactational amenorrhea method knowledge (AOR = 1.5, 95% CI 1.3–4.0). This finding suggests that increasing home to home counseling for family planning by town health extension workers a key mechanism to increase the knowledge gap of LAM for postpartum mothers.

### Conclusions

This study demonstrated that the knowledge of LAM as a contraceptive method was found so low. Our findings reinforce that in order to address the knowledge gap of mothers; improving the uptake of maternal health services and strengthening family planning counseling at their home are a key area for improving the knowledge level of LAM.

## Limitations

This study has some limitation limitations: First, it focuses only on the individual level variables. Factors related to community level such as socio-cultural and family planning program related factors did not address in this study. Second, this study has the usual limitation of a cross sectional study. It is difficult to ascertain the association between knowledge of LAM as a method of contraceptive and the predictor variables since they were measured at one point in time.

## Additional files

**Additional file 1: Figure S1.** Schematic representation of sampling procedure, knowledge of LAM as a contraception method among postpartum women in Aksum town, Tigray Region, northern Ethiopia, June 2015 (n = 604).

**Additional file 2: Table S1.** Description and measurement of variables, Aksum town, Tigray Region, Ethiopia, June 2015.

**Additional file 3: Table S2.** Characteristics of maternal health services use in Aksum town, Tigray Region, northern Ethiopia, June 2015.

## Abbreviations

AOR: adjusted odds ratio
CI: confidence interval
COR: crude odds ratio
EHSTP: Ethiopian Health Sector Transformation Plan
LAM: lactation amenorrhea method
