# Supplementary material for: Knowledge and associated factors of lactational amenorrhea as a contraception method among postpartum women in Aksum town, Tigray Region, Ethiopia
Source: BMC Res Notes. 2018 Sep 3;11:641. doi: 10.1186/s13104-018-3754-2 (PMC6122625; doi:10.1186/s13104-018-3754-2)
Supplement: Supplementary file 1 — Additional file 1: Figure S1. Schematic representation of sampling procedure, knowledge of LAM as a contraception method among postpartum women in Aksum town, Tigray Region, northern Ethiopia, June 2015 (n = 604). [file 13104_2018_3754_MOESM1_ESM.docx]

The two k*ebele* were selected by lottery method

Proportional allocation to size of postpartum women to each  *kebele*

Additional file 1: FigureS1.
