# Supplementary material for: Knowledge and associated factors of lactational amenorrhea as a contraception method among postpartum women in Aksum town, Tigray Region, Ethiopia
Source: BMC Res Notes. 2018 Sep 3;11:641. doi: 10.1186/s13104-018-3754-2 (PMC6122625; doi:10.1186/s13104-018-3754-2)
Supplement: Supplementary file 2 — Additional file 2: Table S1. Description and measurement of variables, Aksum town, Tigray Region, Ethiopia, June 2015. [file 13104_2018_3754_MOESM2_ESM.docx]

**Additional file 2:** Table S1. Description and measurement of variables, Aksum town, Tigray region, Ethiopia, 2015

| Variables | Description and measurement |
| --- | --- |
| Age | Self reported age of respondent at the time of data collection [15-49 years]. |
| Marital status | Marital status of respondent [currently married and un married]. |
| Maternal educational status | Highest level of education level [None educated /Primary; Secondary or higher]. |
| husband educational status | Highest level of education level [None educated /Primary; Secondary or higher]. |
| Maternal occupation | Is the current occupation status categorized: House wife, Government employee, Private employee , Daily Labourer and others* |
| Partner occupation | Is the current occupation status categorized: Government Employee, private employee, and Daily Labourer and others* |
| Institutional delivery | Proportion of women who have given birth at either at health center, hospital or health post |
| Postnatal care utilization | Proportions of women who have postnatal care at least one visits either at health center or hospital. |

***^*^****Farmer, pension or guard*
