# Supplementary material for: Knowledge and associated factors of lactational amenorrhea as a contraception method among postpartum women in Aksum town, Tigray Region, Ethiopia
Source: BMC Res Notes. 2018 Sep 3;11:641. doi: 10.1186/s13104-018-3754-2 (PMC6122625; doi:10.1186/s13104-018-3754-2)
Supplement: Supplementary file 3 — Additional file 3: Table S2. Characteristics of maternal health services use in Aksum town, Tigray Region, northern Ethiopia, June 2015. [file 13104_2018_3754_MOESM3_ESM.docx]

Additional file 3: Table S2.Characteristics of maternal and reproductive health services use in Aksum town, Tigray Region, northern Ethiopia, June 2015

| Variables | Frequency(n) | | Percentage (%) |
| --- | --- | --- | --- |
| Number of abortion |  | |  |
| No abortion | 490 | | 83.1 |
| 1-2 | 95 | | 16.1 |
| 3-4 | 5 | | 0.8 |
| Birth interval |  | |  |
| <24 months | 105 | | 25.2 |
| 24-47 months | 131 | | 31.5 |
| >=48 months | 180 | | 43.3 |
| ANC follow up |  | |  |
| Yes | 579 | | 98.1 |
| No | 11 | | 1.9 |
| ANC visits |  | |  |
| 1-3 | 57 | | 9.4 |
| >=4 | 524 | | 90.6 |
| Place of birth |  | |  |
| Health institution | 577 | | 97.8 |
| Home | 13 | | 2.2 |
| Postpartum period |  | |  |
| 0-12 week | 186 | | 41.5 |
| 13-26 week | 198 | 33.6 | |
| 27-38 week | 141 | 23.9 | |
| 39-52 week | 65 | 11 | |
| PNC |  |  | |
| Yes | 258 | 43.7 | |
| No | 332 | 56.3 | |

**Table 2** Characteristics of maternal and reproductive health services use in Aksum town, Tigray Region, northern Ethiopia, June 2015*(continued)*

| Variables | Frequency(n) | Percentage (%) |
| --- | --- | --- |
| Visited and counseled for FP by HEW in the last 12 months | |  |
| Yes | 457 | 77.5 |
| No | 133 | 22.5 |
| Postpartum menstruation status | |  |
| Menstruating | 205 | 34.8 |
| Amenorrhea | 385 | 65.2 |
| Resumed sexual intercourse | |  |
| Yes | 403 | 68.3 |
| No | 187 | 31.7 |
| LAM knowledge |  |  |
| Yes | 52 | 8.8 |
| No | 538 | 91.2 |

*ANC Antenatal Care*

*PNC Postnatal Care*

*FP Family planning*
